# Overexpression of Phosphoenolpyruvate Carboxykinase Increases Photosynthetic Efficiency and Salt Tolerance in Rice

**DOI:** 10.3390/plants15091402

**Published:** 2026-05-04

**Authors:** Suchismita Prusty, Swetaleena Mishra, Sowmya Poosapati, Durga Madhab Swain, Ranjan Kumar Sahoo

**Affiliations:** 1 School of Biotechnology, Centurion University of Technology and Management, Bhubaneswar 757001, Odisha, India; suchiprusty02@gmail.com (S.P.); swetaleenamishra28@gmail.com (S.M.); 2 Howard Hughes Medical Institute and Plant Molecular and Cellular Biology Laboratory, Salk Institute for Biological Studies, San Diego, CA 92037, USA; 3 Vidya USA Corporation, 7 Otis Stone Hunter Road, Bunnell, FL 32100, USA

**Keywords:** *PEPCK*, salinity stress, antioxidant enzymes, *Oryza sativa*, reactive oxygen species (ROS), photosynthesis

## Abstract

Salinity stress is one of the major obstacles to glycophytic crop production worldwide, including rice. It alters cellular metabolism, causing significant crop destruction that results in substantial reductions in yield. The overexpression of C_4_ enzymes, such as phosphoenolpyruvate carboxykinase (PEPCK), at high levels in C_3_ transgenic plants through genetic engineering can decrease oxidative stress while increasing photosynthetic capabilities. In this research, we evaluate the efficiency of transgenic rice plants (*Oryza sativa* L. cv. IR64) overexpressing *PEPCK* genes in mitigating salinity stress and increasing photosynthetic efficiency. The T1 transgenics showed increased levels of several biochemical factors, including ascorbate peroxidase (APX), catalase (CAT), proline, glutathione reductase (GR), and guaiacol peroxidase (GPX) activities. This was accompanied by reduced levels of malondialdehyde (MDA), hydrogen peroxide (H_2_O_2_), and electrolytic leakage, suggesting an effective antioxidant defense mechanism against the oxidative damage driven by salt stress. Photosynthetic parameters—such as chlorophyll content, net photosynthetic rate, intercellular CO_2_ content, and stomatal conductance—were elevated in transgenic plants compared to control plants. The transgenics also exhibited superior agronomic characteristics. Our findings provide conclusive evidence of the *PEPCK* gene’s potential role in regulating salt stress response and tolerance in rice plants.

## 1. Introduction

The rapid expansion of the global population has led to a significant increase in the demand for food production, despite a projected decline in available fertile land. To meet the needs of an estimated population of 9.7 billion by 2050, it is projected that a 70% increase over current production levels will be required, particularly for staple crops such as rice (*O. sativa*), wheat (*Triticum aestivum*), soy (*Glycine max*), and maize (*Zea mays*) [1]. Furthermore, natural catastrophes and shifting climatic conditions present major obstacles to achieving target crop yields. One of the primary constraints affecting global agricultural productivity is soil salinity, which affects approximately 25% of total arable land. In certain regions, such as Central Asia, salinity afflicts 60–65% of the soil in stress-affected areas [2]. These conditions lead to a significant 10–25% reduction in crop yields and, in extreme instances, can result in total desertification [3].

Excessive salt concentrations (100–200 mM NaCl) inhibit the growth of major glycophytic crops, including rice, corn, wheat, soybeans, potatoes, and various legumes. Rice (*O. sativa* L.), a primary food source for over half the global population, is notably salt-sensitive. For instance, soil salinity results in rice output deficits of as much as 45% in the Indo-Gangetic Basin of India and 36–69% in the Indus Basin of Pakistan. Consequently, immediate intervention to mitigate the effects of salinity is essential for maintaining cropland viability and boosting productivity in an economically sustainable manner. To establish long-term food security, researchers are increasingly focused on developing new transgenic varieties, as the introduction of genetically engineered crops with enhanced salt tolerance represents a practical and necessary solution to the current crisis.

One possible strategy involves the integration of the C_4_-like mechanism into C_3_ plant mesophyll cells [4]. The transformation from C_3_ to C_4_ plants requires gradual modifications in metabolic pathways that might lead to the development of improved varieties with evolutionary advantages. Although the engineering of high-level and cell-specific gene expression poses obstacles for the insertion of C_4_ biochemistry into C_3_ plants, genes encoding the majority of metabolite transporters and enzymes of the C_4_ pathway have recently been discovered [5]. One such gene with significant potential is *PEPCK*, which is engaged in several metabolic activities, justifying its inclusion in the current study.

PEPCK functions as a primary cytosolic decarboxylase enzyme present in C_4_ plants and has been observed to respond positively to salt stress in some plant species [6]. *PEPCK* has a role in gluconeogenesis and the TCA cycle [7]. It also contributes to the maintenance of pH and is involved in the metabolism of amino acids, nitrogen, sugars, organic acids, and malate [8]. Through genetic engineering, transgenic crops expressing high levels of the C_4_ enzymes PEPC or PEPCK have demonstrated improved photosynthetic capability [8]. Although previous studies have reported the involvement of *PEPCK* in response to drought stress and various environmental factors, limited data exist regarding its involvement in salt stress tolerance, especially in C_3_ crops like rice, which require detailed analysis [9].

In the present research, it is postulated that *PEPCK* gene overexpression in rice might enhance carbon metabolism and improve photosynthetic efficacy, even under salinity stress. To validate this, we overexpressed the *PEPCK* gene to generate transgenic rice plants and analyzed their genetic, physiological, biochemical, and agronomic responses under salt stress, while also exploring the underlying mechanisms. This study aims to overexpress a gene (*PEPCK*) from a C_4_ plant species in a C_3_ plant system to enhance photosynthetic performance and abiotic stress tolerance, with the goal of inducing C_4_-like physiological characteristics. The findings of our research could further contribute to our understanding of C_4_-linked metabolic attributes and support current attempts to increase the photosynthetic efficiency of rice.

## 2. Materials and Method

### 2.1. Generation and Molecular Characterization of Transgenic Rice Plants Overexpressing PEPCK Gene

The coding region of the *PEPCK* gene was amplified from *Urochloa panicoides* cDNA (GenBank accession no. AF136163.1) using the forward primer 5′-ATGGAGTTGGTTCAGAATAAAA-3′ and the reverse primer 5′-GGTGTGGAGTTCTCTTA-3′. The amplified fragment was cloned into pRT-100 at the NcoI site to generate the 35S promoter:*PEPCK*:poly(A) cassette, which was subsequently subcloned into the PstI site of pCAMBIA1301. The construct was introduced into embryogenic calli derived from mature rice seeds (*O. sativa* L. cv. IR64) via *Agrobacterium tumefaciens* strain LBA4404 following Sahoo and Tuteja [10]. Genomic DNA (0.15–0.20 µg) was isolated from the fresh leaves (0.5 g) of putative T_1_ transgenic plants and used as a template for PCR analysis. Screening was performed using a forward primer (5′-ATGGAGTTGGTTCAGAATAAAA-3′) and a reverse primer (5′-GGTGTGGAGTTCTCTTA-3′) to confirm the presence of the transgene. Transgene integration was further verified by Southern blot analysis as described by Sambrook et al. [11]. Moreover, 20 µg of genomic DNA was digested with XbaI and separated on 0.8% agarose gels by gel electrophoresis. It was then subjected to depurination (0.2 N HCl) and denaturation (1.5 M NaCl, 0.5 N NaOH), followed by a neutralization process (0.5 M Tris-HCl, 1.5 M NaCl, pH 8.0) wherein each step was carried out for 20 min. The DNA was transferred to a nylon membrane through capillary blotting (6× SSC buffer and 0.4 N NaOH) overnight and crosslinked with UV-Crosslinkers after being neutralized in 1 M Tris Cl. for 10 min. The membrane was pre-hybridized in buffer containing 5× SSC, 5× Denhardt’s solution, 0.1% SDS, 100 µg/mL denatured salmon sperm DNA, and 10% dextran sulfate at 60 °C. Hybridization was performed with a denatured, labeled DNA probe (α−^32^P dCTP-labeled CaMV 35S promoter fragment) for 16–18 h at 60 °C. Post-hybridization washes were carried out under stringent conditions, and signals were detected by autoradiography.

The wild type (non-transformed) plants served as controls. Ten plants per independent line (three biological replicates and ten technical replicates, for a total of thirty plants) were used as biological replicates. Analyses were performed on T_1_ seedlings subjected to stress treatments.

### 2.2. Tolerance Index Assessment of T_1_ Plants

Three-week-old IR64 T_1_ rice plants overexpressing *PEPCK* and wild-type (control) plants were grown in one big tank filled with 200 mM NaCl instead of water. The response of these plants was recorded in 30-day intervals as described in our earlier studies [12,13]. The salt level was ensured in the course of the experiment periodically by maintaining a fixed volume of solution and adding more NaCl solution as required. Despite the fact that 200 mM NaCl level is higher than usual field salinity, this metric is frequently used in controlled experiments in order to identify stress tolerance thresholds and assess the performance of transgenic lines under harsh saline circumstances. Following the stress treatment, various physiological and biochemical assays were performed to evaluate the stress response, including measurements of photosynthetic efficiency, growth parameters, and stress-related biochemical markers. Each treatment consisted of three biological replicates, with at least 8–10 plants per replicate.

The tolerance index (TI) was calculated for *PEPCK* T_1_ transgenic lines (L2, L7, and L12) and wild-type plants under salt stress using the formula:

T_1_ (%) = [(plant dry weight under 200 mM NaCl)/(plant dry weight under water)] × 100

### 2.3. Relative Gene Expression Analysis

For gene expression studies, total RNA was extracted from leaf tissues after 24 h of treatment (the time frame helps to record early transcriptional reactions during the salt stress acclimation period, before serious stress-induced damage begins), using TRIzol reagent. First-strand cDNA was synthesized using SuperScript II Reverse Transcriptase (Invitrogen, Carlsbad, CA, USA) and an oligo(dT)_18_ primer according to the manufacturer’s instructions. Quantitative real-time PCR (qRT-PCR) was performed using gene-specific primers (Forward: 5′-GGAAATCCTCGACCCCATCA-3′; Reverse: 5′-CGATCTTGTAGCTGGCGAAC-3′). The relative levels of the transcript accumulated for the gene were normalized to α-tubulin (primers: forward 5′-GGTG GAGGTGATGATGCTTT-3′ and reverse 5′-ACCACGGGCAAAGTTG TTAG-3′). Amplification was carried out in a StepOne Real-Time PCR System (Applied Biosystems, Foster City, CA, USA) under the following conditions: 95 °C for 30 s, 60 °C for 30 s, and 72 °C for 30 s. Relative gene expression was quantified using the 2^−ΔΔCT^ method as described by Jayaraman et al. [14]. The PCR efficiency, which is dependent on the assay, performance of the master mix and quality of the sample, was calculated as efficiency = 10 (–1/slope) − 1 (3.6C slope C 3.1) by the software itself (Applied Biosystems, StepOne™ Software v2.3, http://www.appliedbiosystems.com). ‘C’ is defined as threshold cycle. All reactions were performed in triplicate, and mean expression values were calculated for each sample.

### 2.4. Measurement of Salinity Tolerance by Leaf Disk Senescence Assay

Leaf disks (1 cm × 1 cm) were excised from fully expanded leaves of three-week-old IR64 T_1_ rice plants overexpressing *PEPCK* (lines L2, L7, and L12) and wild-type control plants. The disks were floated in NaCl solutions containing 100 mM and 200 mM for 72 h (because it enables the formation of quantifiable salt-induced senescence without causing total tissue degeneration) at room temperature. Control disks were incubated in distilled water under identical conditions. After the treatment, chlorophyll retention and the degree of leaf bleaching were assessed to evaluate salt stress tolerance. The experiment was conducted with three biological replicates following the method described by Tuteja et al. [12]. Using spectrophotometry (wavelengths—A_663_, A_645_), the total chlorophyll (Ca + b) content was calculated. After the 300 mg of leaves were ground into a powder using liquid nitrogen, they were put into a 15 mL Falcon tube. The tube was stirred after adding 5 mL of 80% acetone, then left in the dark for the entire night. The centrifugation process ran for 15 min at 4 °C (3000 rpm). The supernatant was moved to a fresh centrifuge tube, and spectrophotometry was used to determine the absorbance of chlorophyll (henceforth abbreviated as A). The following formula is used to compute the chlorophyll concentrations (80% acetone was used as a blank control).

Ca + b (mg/g) = [8.02 × A_663_ + 20.20 × A_645_] × V/1000 × W

where V = volume of the extract (mL); W = weight of fresh leaves (g).

### 2.5. Assessments of Antioxidants in PEPCK Transgenic Lines

The 21-day-old control plant and *PEPCK* transgenic plant seedlings were cultivated in 200 mM NaCl for 24 h in this experiment before being utilized for biochemical studies. Following the salt stress treatment, the accumulation of malondialdehyde (MDA), hydrogen peroxide (H_2_O_2_), as well as ion leakage were analyzed [15]. The enzyme activity of the ascorbate peroxidase (APX), catalase (CAT), guaiacol peroxidase (GPX), and glutathione reductase (GR) was also evaluated, as they play significant functions in stress responses [15].

#### 2.5.1. Proline Estimation

Proline content was determined following the method of Bates et al. Fresh root tissues (500 mg) were ground to a fine powder in liquid nitrogen and homogenized in 10 mL of 3% sulphosalicylic acid under ice-cold conditions [16]. After centrifuging the resulting mixture at 10,000× *g* for 15 min, 2 mL of the solution was combined with 2 mL of acid ninhydrin and glacial acetic acid. The mix was cooled in ice to stop the chemical reaction after being incubated at 100 °C for one h, during which time a colorful complex was produced in the water bath. The colored complex was vortexed for 15–20 s after adding 4 mL of toluene. Afterward, at 520 nm, the optical density of the layer comprising the chromophore was evaluated to determine the proline content by utilizing an L-Proline standard curve.

#### 2.5.2. Determination of H_2_O_2_ Content

The H_2_O_2_ level was determined using an updated version of Jana and Choudhuri’s approach [17]. Hydrogen peroxide (H_2_O_2_) content was determined using fresh leaf tissue (100 mg). The tissue was ground to a fine powder in liquid nitrogen and homogenized in 3 mL of 50 mM phosphate buffer (pH 7.0). The homogenate was filtered and centrifuged at 6000× *g* for 25 min at 4 °C. An aliquot of 0.9 cm^3^ of the supernatant was mixed with 0.3 cm^3^ of 1% (*v*/*v*) TiCl_4_ in concentrated HCl, and the mixture was centrifuged again at 6000× *g* for 15 min at 4 °C for H_2_O_2_ estimation. At 410 nm, the absorbance of yellow supernatant was determined. The values were compared with a standard curve.

The estimation of the levels of electrolytic leakage, lipid peroxidation, and relative water content (RWC) was determined using the procedure outlined by Tuteja et al. [13].

#### 2.5.3. Lipid Peroxidation Measurement (MDA Content)

Malondialdehyde (MDA), a breakdown product of lipid peroxidation, was measured to quantify lipid peroxidation [18]. Using a mixer mill (MM400, Retsch, Haan, Germany) with two cycles of 35 Hz per minute, leaves (0.1 g) were crushed into a fine powder. Following the addition of 1 mL of 0.1% trichloroacetic acid (TCA), the resulting solution was centrifuged for 15 min at 5000× *g* and 25 °C. Following centrifugation, 0.75 mL of 0.25% 2-thiobarbituric acid in 10% TCA was mixed with 0.3 mL of the supernatant, and the absorbance was measured at 532 nm and 600 nm. Implementing an absorption coefficient of 155 mM^−1^ cm^−1^, the MDA concentration was evaluated by deducting the absorbance of the supernatant at 600 nm from that at 532 nm.

#### 2.5.4. Electrolytic Leakage (Membrane Permeability)

Three days post salt stress, leaves were cut into 1 cm square pieces, placed in a test tube, and rinsed with 5 mL of deionized water to remove surface contaminants. Electrical conductivity (EC) was subsequently evaluated both prior to and after autoclaving at 121 °C for 20 min, while the material was immersed in 5 mL of deionized water in a test tube for two hours.

The formula for calculating the cell membrane stability [%] was 100 − [(EC1/EC2) × 100], where EC1 represents the electric conductivity following a two-hour dip in deionized water and EC2 represents electrical conductivity following a 20 min autoclave [19].

#### 2.5.5. Relative Water Content

Barrs and Whetherley’s approach was implemented, and plants from all treatments were chosen at random [20]. To calculate initial mass (Mi), a leaf specimen weighing about 0.1 g was divided into smaller fragments and analyzed. To calculate the completely water-saturated mass (Mf), the leaf specimens were submerged in de-ionized water for 12 h. After a three-day drying at 60 °C, the specimen’s dry mass (Md) was measured, and RWC [%] was calculated using the formula [(Mi − Md)/(Mf − Md)] × 100.

### 2.6. Quantification of Photosynthetic Parameters

Over the course of 30 days (perfect to access developmental and metabolic responses, so they will have enough time to acclimate and develop stable phenotypic differences), mature IR64 rice control plants and *PEPCK* transgenic rice plants were subjected to 0 mM and 200 mM NaCl, respectively. On a sunny weather between 10:00 a.m. and 12:00 p.m., the fourth and fifth fully extended leaves of transgenic lines (L2, L7, and L12) as well as control plants were measured for stomatal conductance (gs), intercellular CO_2_ concentration (Ci), net photosynthetic rate (Pn), and transpiration rate using an infrared gas analyzer (IRGA from LiCor, located in Lincoln, NE, USA). The examination was conducted in the following atmospheric factors: atmospheric temperature of 30 ± 2 °C, relative humidity of 68.2 ± 6%, atmospheric CO_2_ of 404 μmol mol^−1^, photosynthetically active radiation (PAR) of 1900 ± 6 μmol m^−2^ s^−1^.

### 2.7. The Agronomic Attributes of T_1_ Transgenic Plant

Following a 30-day treatment with 100 mM NaCl and 200 mM NaCl, various growth related agronomic parameters were measured (done on 60 day old plant, record taken at the end of the treatment period), including plant height, leaf area, number of tillers/plant, root length, root dry weight, straw dry weight, plant dry weight pre- and post-salt stress in both control and *PEPCK* transgenic rice lines. Yield-related agronomic parameters (90 day old plant, without further treatment with salt stress) such as plant height, number of tillers/plant, number of panicles/plant, number of chaffy grains/panicle, number of filled grain/panicle and, leaf area, 100-grain weight, root dry weight, straw dry weight, root length, and plant dry weight pre- and post-salt stress in both control and *PEPCK* transgenic rice lines were also measured and on a meter scale, the length of the shoot and roots were determined. Plant samples were desiccated in a hot-air oven (Memmert, Model 500, Schwabach, Germany) at 80 °C for four days until a uniform weight was achieved. Dry weight was established by incubating the specimens in a desiccator. The leaf area was determined using a leaf area meter (manufactured by Systronics in Hyderabad, India).

### 2.8. Determining the Endogenous Ion Content and Soluble Sugar and Hormones

To estimate endogenous ions (potassium, nitrogen, sodium, and phosphorus concentration), leaves from T_1_ transgenic lines and control lines cultivated for 8 weeks (to ensure that plants under prolonged exposure to salt attained a stable vegetative stage, allowing for accurate assessment of long-term ion buildup and homeostatic regulation in both control and transgenic lines) on 0 mM NaCl and 200 mM NaCl, respectively, were used. Jackson’s method was utilized to calculate the total nitrogen concentration [21]. A spectrophotometer was utilized to determine the phosphorus concentration in accordance with Gupta’s instructions [22]. Using a flame ionization photometer and regular procedure, the potassium content was determined [23]. The Munns et al. [24] technique was used to test the sodium content. After subjecting both transgenic and control plants to a 24 h salt treatment, the amounts of fructose and glucose in both the roots and shoots were determined [25]. As previously stated by Chen et al. [26], estimates of the endogenous plant hormones (GA and IAA) have been determined. Zeatin was measured utilizing optimized MRM transitions and genuine standards under identical analytical circumstances. Approximately 1 g of the leaf (fresh leaves) was collected, frozen instantly in liquid nitrogen and homogenized to fine powder, out of which 100 mg of powdered tissue was used for hormone extractions. A total of 1 mL of cold 80% (*v*/*v*) methanol was used to derive the phytohormones, which then underwent incubation for 12 h at 4 °C with occasional mixing. It was then centrifuged at 12,000× *g* (4 °C for 15 min), and the supernatant was collected and dried completely using a stream of nitrogen gas (35 °C). The dry supernatant was dissolved in 100 mL of acetonitrile (95% *v*/*v*) and re-centrifuged for 15 min at 12,000× *g* (4 °C). The supernatant was acquired for the assessment of liquid chromatography–mass spectrometry. Quality control samples were prepared from mixing all experimental samples to acquire a representative composite sample. This was used successively (once after every 10 samples) to ensure the integrity of the results. The hormones were determined by liquid chromatography–electrospray ionization tandem mass spectrometry (LC–ESI–MS/MS) system.

Experimental conditions—HPLC: column, Waters ACQUITY UPLC HSS T3 C18 (1.8 μm, 2.1 mm × 100 mm); solvent system: water (0.1% acetic acid): acetonitrile (0.1% acetic acid). The gradient conditions—100:0 *v*/*v* *V*/*V* at 0 min, 5:95 *v*/*v* at 11.0 min, 5:95 *v*/*v* at 12.0 min, 95:5 *v*/*v* at 12.1 min, and 95:5 *v*/*v* at 15.0 min; flow rate: 0.40 mL/min; temperature: 40 °C. The injection volume—5 μL. A triple quadrupole-linear ion trap mass spectrometer (Q TRAP, API 4500, Q TRAP LC/MS/MS System, AB Sciex software-SCIEX OS version 3.4.5) having an electrospray ionization (ESI) source working with positive ion mode was used for the mass spectrometric study. ESI source parameters—ion source: turbo spray; source temperature: 550 °C; ion spray voltage (IS): 5500 V; ion source gas I (GSI), gas II (GSII), and curtain gas (CUR) were set at 55, 60, and 25.0 psi, respectively, with high collision gas (CAD). Multiple reaction monitoring (MRM) mode was used to collect data, and each hormone’s transitions were calibrated, using nitrogen (5 psi) as the collision gas. Hormones were determined by comparing their mass transitions and retention durations to those of real standards. External calibration curves were used for quantification, and outcomes were reported as ng g^−1^ dry weight. The hormones were characterized by their distinct retention times and MRM transitions that were derived from verified standards under the same LC–MS/MS conditions. The detection limit lies between 0.1 and 1.0 ng g^−1^.

### 2.9. Statistical Analysis

Data from three independent transgenic rice lines were collected, and mean values and standard errors were calculated. Applying SPSS (12.0 Inc., Chicago, IL, USA), the ANOVA test was run on the collected data to find the least significant difference (LSD) for the statistically significant data, which allowed for the identification of treatment-wise changes in the mean. Duncan’s multiple-range analyses (DMRT) were utilized to determine the means.

## 3. Results

### 3.1. Molecular Assessment of the Transgenic PEPCK Plants

**Figure 1:**
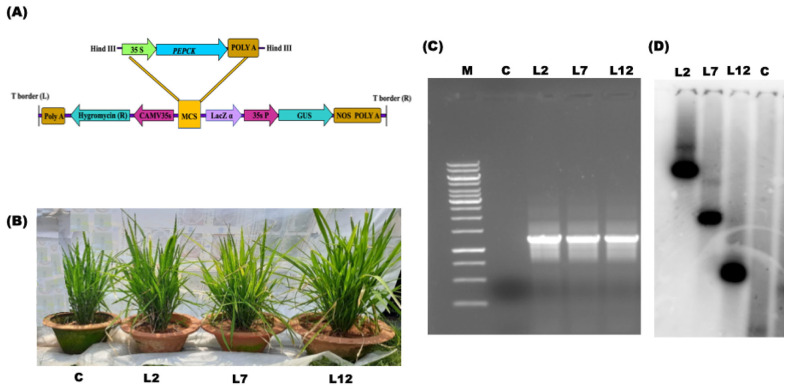
Molecular characterization and analysis of the expression of T_1_ transgenic lines (*PEPCK*). (**A**) Schematic representation of T-DNA region of pCAMBIA1301, containing the *PEPCK* gene (1.4 kb) inserted in *HindIII* restriction enzyme site of the Multiple Cloning Site (MCS) with the promoter (CaMV35S) and terminator (poly A). (**B**) The *PEPCK* overexpressing T_1_ transgenic lines (L2, L7, and L12) and control plant (wild type) were used for further analysis (60-day-old plants). (**C**) Polymerase chain reaction (PCR) analysis of *PEPCK* overexpressing transgenic (T_1_) lines by using CaMV35S promoter-specific forward and gene reverse primers, showing the expected amplification of a 1.4 kb fragment in three independent transgenic lines (L2, L7, and L12). Here, M represents the marker, and L2, L7 and L12 are independent transgenic lines. (**D**) Southern blot analysis showing the integration and copy number of the *PEPCK* gene in all three transgenic lines.

**Figure 2:**
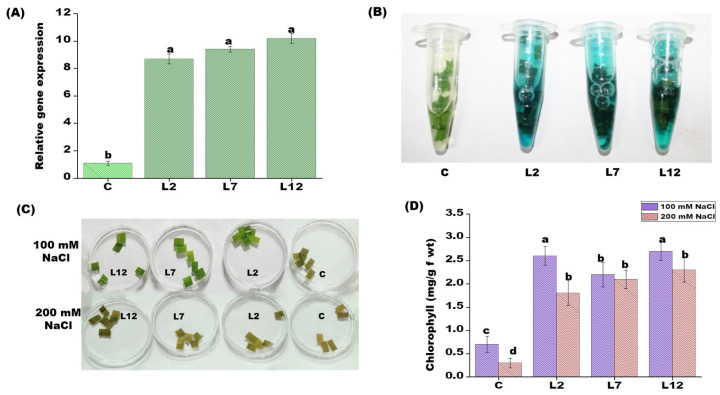
Genotypic and phenotypic characterization of *PEPCK* overexpressed T_1_ transgenic lines. (**A**) Relative gene expression analysis of the T_1_ *PEPCK* transgenic lines to observe the RNA expression (fold change) in control and transgenic lines. (**B**) Visualization of GUS activity in leaf tissues of transgenic lines with control plants. (**C**) Leaf disk senescence assay for salt tolerance in T_1_ *PEPCK* transgenic rice lines with control plants (after 72 h of salt treatment). (**D**) Chlorophyll content (mg g^−1^ fw) in T_1_ *PEPCK* transgenic lines under 100 and 200 mM NaCl after 72 h. Each value represents the mean of three replicates ± SE. Different letters on the top of bars indicate significant differences at *p* ≤ 0.05 level as determined by Duncan’s multiple range test (DMRT).

Transgenic IR64 rice plants were generated using the pCAMBIA1301-*PEPCK* T-DNA construct (Figure 1A). The presence of the *PEPCK* transgene was confirmed via PCR using gene-specific primers and β-glucuronidase (GUS) expression analysis. Three transgenic T_1_ lines (L2, L7, and L12) were selected based on positive GUS staining and PCR results (Figure 1B–D and Figure 2B). Quantitative real-time PCR (qRT-PCR) revealed that these selected lines exhibited approximately a 10-fold increase in *PEPCK* transcript levels compared to control plants grown under normal conditions (Figure 2A). The stable integration of the *PEPCK* transgene was confirmed through Southern blot analysis, where the lines L2 and L7 show two distinct bands, suggesting multiple copy insertion, and L12 shows one band, suggesting one copy number.

### 3.2. PEPCK Overexpression Confers Salt Tolerance

Leaf disk assay was conducted as an initial screen to evaluate the salt stress tolerance of T_1_ transgenic lines (L2, L7, and L12) overexpressing *PEPCK*. Leaf disks (approximately 1 cm × 1 cm) from transgenic and control plants were incubated in 100 and 200 mM NaCl for 72 h. Following salt treatment, leaf tissues exhibited visible discoloration. Compared to control plants, all transgenic lines showed a significantly lower reduction in chlorophyll content, suggesting enhanced tolerance to saline stress at both mild (100 mM) and severe (200 mM) concentrations (Figure 2C,D). Within the transgenic lines, L7 showed maximum chlorophyll retention under 100 mM NaCl and 200 mM NaCl, followed by L12 and L2 (Figure 2C,D).

### 3.3. Biochemical and Physiological Responses of PEPCK Transgenic Plants Under Salt Stress

**Figure 3:**
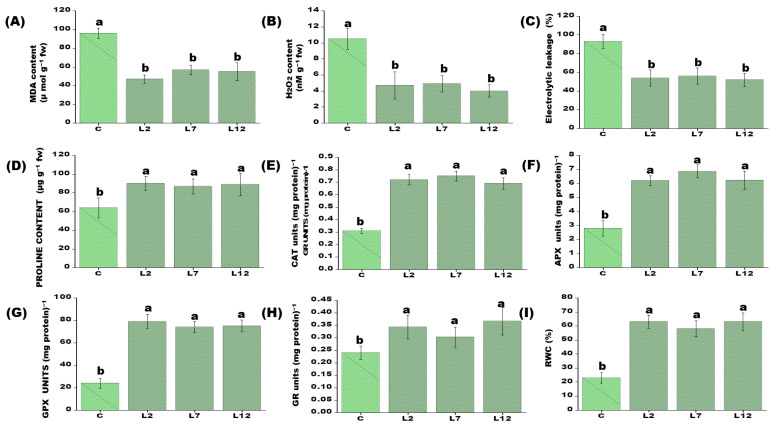
Biochemical analysis and the response of the antioxidant machinery in *PEPCK* overexpressing T_1_ transgenic lines (L2, L7, and L12) and control rice plants exposed to 24 h salt stress (200 mM NaCl). (**A**) Determination of lipid peroxidation expressed in terms of MDA content. (**B**) Changes in the level of hydrogen peroxide (H_2_O_2_) content. (**C**) Measurement of electrolytic leakage. (**D**) Changes in the level of proline accumulation. (**E**) Catalase (CAT) activity in transgenic lines after salt stress, where one unit of enzyme activity is defined as 1 μmol H_2_O_2_ oxidized min^−1^. (**F**) Changes in ascorbate peroxidase (APX) enzyme activity in transgenic lines after salt stress, where one unit of enzyme activity is defined as 1 μmol of ascorbate oxidized min^−1^. (**G**) Changes in guaiacol peroxidase (GPX) activity in transgenic lines after salt stress. (**H**) Changes in glutathione reductase (GR) activity in transgenic lines after salt stress, where one unit of enzyme activity is defined as 1 μmol of GS-TNB formed min^−1^ due to reduction in 5,5′-dithiobis-(2-nitrobenzoic acid), known as DTNB. (**I**) Estimation of relative water content (RWC%) in leaf disks of transgenic and control rice. Each value represents the mean of three replicates ± SE. Different letters on the top of bars indicate significant differences at *p* ≤ 0.05 level as determined by Duncan’s multiple range test (DMRT).

To further evaluate physiological responses to salt stress, selected T_1_ transgenic lines were subjected to severe salinity (200 mM NaCl). Following treatment, the transgenic plants exhibited two-fold lower MDA levels compared to water-treated controls. Additionally, *PEPCK*-overexpressing lines showed approximately 2.5-fold lower H_2_O_2_ accumulation and 1.5-fold lower ion leakage after salt exposure (Figure 3A–C). The enhanced salt tolerance was further supported by a robust biochemical defense response. Under 200 mM NaCl stress, the transgenic lines displayed a 1.5-fold increase in proline content and a 2.7-fold higher RWC. Furthermore, the activities of key antioxidant enzymes—including CAT (~1.5-fold), APX (~2.4-fold), GPX (~3.2-fold), and GR (~2.4-fold)—were substantially upregulated in the transgenic lines compared with control plants.

### 3.4. PEPCK Alleviates Agronomic Features in Rice Under Salt Stress

**Figure 4:**
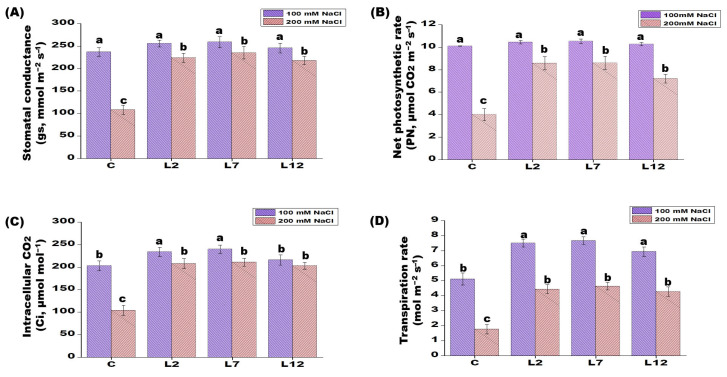
Measurement of photosynthetic characteristics of control and *PEPCK* T_1_ transgenic lines (L2, L7, and L12) under 0 mM NaCl and 200 mM NaCl treatment. (**A**) Stomatal conductance. (**B**) Net Photosynthetic rate. (**C**) Intracellular CO_2_. (**D**) Transpiration rate. Values are means of three replicates ± SE (n = 3). Different letters on the top of bars indicate significant differences at *p* ≤ 0.05 level as determined by Duncan’s multiple range test (DMRT).

To assess the effect of salt stress on the growth of rice lines overexpressing *PEPCK*, several agronomic and physiological parameters were measured, including plant height, tiller number per plant, panicle number per plant, filled grains per panicle, chaffy grains per panicle, straw dry weight, 100-grain weight, root length, root dry weight, leaf area, and shoot length (Table 1 and Table 2). The T_1_ *PEPCK* overexpressing lines (L2, L7, and L12) exhibited a segregating ratio of 3:1 (HygR:HygS), approximately, as observed in the T_1_ transgenic rice seeds (Table 3). While comparing the developmental rate of T_1_ transgenics rice seedlings under NaCl stress to that of control plants, no discernible variation was seen. Under salt stress, T_1_ transgenic plants exhibited higher values for most growth parameters compared with control plants. In addition, photosynthetic parameters—including stomatal conductance (gs), Pn, Ci, and transpiration rate—were significantly elevated in the transgenic lines relative to controls. Additionally, among the transgenic lines, L7 showed higher photosynthetic parameters, followed by L12 and L2 (Figure 4A–D). Notably, control rice plants failed to reach the flowering stage under salt stress conditions (Table 2 and Table 3). Furthermore, ion content analysis under 200 mM NaCl revealed that *PEPCK* T_1_ transgenic lines accumulated lower sodium levels than control plants, while exhibiting higher concentrations of potassium, phosphorus, and nitrogen (Table 2). Here, too, L2 transgenic lines showed the lowest response, and L7 showed the highest response. These results indicate that *PEPCK*-overexpressing plants experienced reduced stress and were able to maintain normal physiological and growth activities under saline conditions.

### 3.5. Carbohydrate Metabolism and Hormone Signaling Under Salt Stress

**Figure 5:**
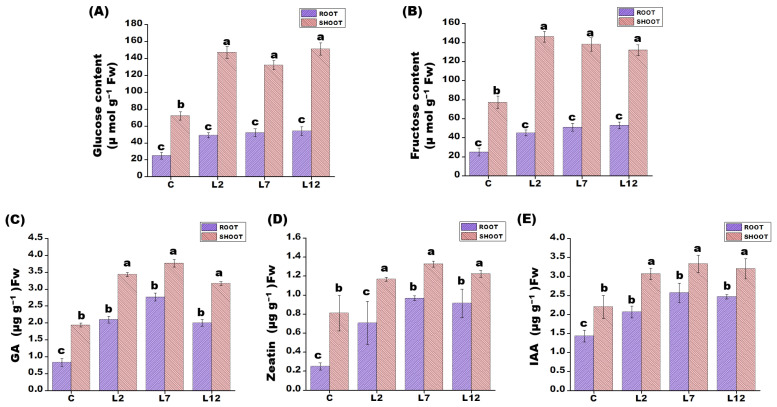
Soluble sugar and endogenous hormone content in roots and shoots of *PEPCK* overexpressing T_1_ transgenic lines (L2, L7, and L12) compared to control rice plants exposed to 24 h salinity stress (200 mM NaCl). (**A**) Glucose content in shoots and roots in transgenic lines and control line after salt stress. (**B**) Fructose content in shoots and roots in transgenic lines and control line after salt stress. (**C**) Endogenous GA content in transgenic lines and control line after salt stress. (**D**) Endogenous Zeatin content in transgenic lines and control line after salt stress. (**E**) Endogenous IAA content in transgenic lines and control line after salt stress. Each value represents the mean of three replicates ± SE. Different letters on the top of bars indicate significant differences at *p* ≤ 0.05 level as determined by Duncan’s multiple range test (DMRT).

During salt stress, *PEPCK*-overexpressing T_1_ plants accumulated significantly higher levels of soluble sugars compared with control rice lines. Specifically, fructose levels increased by approximately 2.5-fold and glucose levels by nearly 2-fold in both shoots and roots of the transgenic plants (Figure 5A,B). In addition to changes in carbohydrate metabolism, hormone profiling revealed notable differences between transgenic and control plants. The roots and shoots of *PEPCK*-overexpressing lines exhibited higher concentrations of key growth-regulating phytohormones, including (GA), zeatin, and (IAA), compared with control plants (Figure 5C–E).

## 4. Discussion and Conclusions

Rice production is severely affected by salinity, a complex multigenic stress that influences multiple physiological and metabolic processes in plants. Previous studies have reported the involvement of *PEPCK* in plant tolerance to abiotic stresses, including salinity and drought, in species such as *Arabidopsis thaliana* and *Sorghum bicolor* [27,28]. Tolerance to salt stress requires the coordinated regulation of several processes, including ion homeostasis and reactive oxygen species (ROS) detoxification [29]. Therefore, the objective of the present study was to investigate the role and possible mechanism of *PEPCK* in conferring salt stress tolerance in rice (*O. sativa* L. cv. IR64).

Compared with other abiotic stresses, *PEPCK* expression has been reported to increase nearly three-fold under NaCl treatment. Previous studies have also shown that several genes, including *OsHKT1*, *PDH45* and *OsBAT1*, are activated in response to salt stress [13,30]. In the present study, transgenic rice lines overexpressing *PEPCK* were generated, and three representative lines (L2, L7, and L12) were selected for functional validation under saline conditions.

A decline in chlorophyll content under abiotic stress is often associated with chlorophyll degradation, which has been widely reported in various crops, including rice [31]. Using the salt tolerance index and the leaf disk senescence assay, we observed significantly enhanced tolerance to salinity in *PEPCK*-overexpressing transgenic rice lines. Exposure to salt stress (100 and 200 mM NaCl) caused visible leaf bleaching in both transgenic and control plants, reflecting stress-induced damage (Figure 2C,D). However, the loss of chlorophyll was more pronounced at 200 mM NaCl than at 100 mM NaCl, and the degree of bleaching was greatest in the control plants. In contrast, the transgenic lines exhibited enhanced tolerance under both moderate (100 mM) and severe (200 mM) salinity conditions. Under normal growth conditions, the transgenic plants showed development comparable to control plants, whereas under salt stress, they exhibited significantly better growth, demonstrating the beneficial effect of *PEPCK* overexpression.

Furthermore, the transgenic lines maintained higher endogenous nutrient levels, indicating an improved capacity to cope with salt stress. Our findings perfectly aligned with the findings of Abdala et al. [32] in their research on *O. sativa* L. spp. Japonica, var. Nipponbare, where the enhanced salt tolerance in the transgenic lines was associated with improved endogenous nutrient levels due to expression of stress-responsive genes like *AP59*, *AP37*, and *DREB1A*. Similar observations have been reported previously where overexpression of *OsSTAP1* in rice was shown to enhance salt tolerance by improving ion homeostasis [33]; however, the effect on soluble sugar accumulation was not examined in that study. Under salt stress, leaves of the *PEPCK*-overexpressing transgenic lines accumulated higher potassium and lower sodium levels compared with control plants. These results suggest that *PEPCK* overexpression may restrict sodium accumulation in leaves, thereby protecting the photosynthetic machinery from salt-induced damage.

The *PEPCK* transgenic lines maintained significantly higher chlorophyll content under saline conditions compared with control plants, which could be linked to the increased activity of antioxidant enzymes, ROS detoxification and lower H_2_O_2_ levels. These findings were consistent with earlier reports on improved stress tolerance in transgenic rice crops [30,31].

The presence of higher levels of chlorophyll content in *PEPCK* transgenic lines under saline conditions implies increased protection of the photosynthetic system. This impact may be attributable to improved antioxidant enzyme activity and effective ROS detoxification, resulting in decreased H_2_O_2_ buildup and maintenance of chloroplast integrity. *OsBAT1* and *PDH45* genes overexpressed in transgenic rice plants with enhanced photosynthetic efficiency and stress tolerance had proven to retain chlorophyll under stress in a comparable manner, indicating the protective function of metabolic and redox control [30,31]. However, these studies did not detect substantial changes in sugar buildup, suggesting that *PEPCK* may be involved in controlling carbon partitioning and osmotic balance under stressful circumstances. In support of our study, the suppression of *OsNYC3*, the overexpression of chloroplastic FBPase, and the occurrence of drought-tolerance QTLs have been linked to increased chlorophyll retention under stress conditions [34,35,36]. Salt stress typically reduces photosynthetic parameters, such as intercellular CO_2_ concentration (Ci), net photosynthetic rate (Pn), and stomatal conductance (gs). However, the reduction in these parameters was less pronounced in the *PEPCK*-overexpressing lines than in the control plants, indicating improved photosynthetic performance under salinity stress. These findings are consistent with earlier studies reporting enhanced stress tolerance in transgenic plants [31,37]. The ability of the transgenic plants to maintain higher chlorophyll levels likely contributed to improved regulation of the photosynthetic system during salt stress.

Salt stress is known to induce the production of reactive oxygen species (ROS), which can damage proteins, nucleic acids, mitochondria, chloroplasts, and plasma membranes through lipid peroxidation and oxidative degradation [38]. In the present study, transgenic plants exhibited significantly lower levels of lipid peroxidation, ion leakage, and H_2_O_2_ accumulation compared with control plants under salt stress. These results are consistent with findings from previous studies [39,40]. Salinity-induced accumulation of H_2_O_2_, a major ROS, can oxidatively damage biomolecules such as proteins, lipids, and nucleic acids, leading to loss of membrane integrity [41]. Plants mitigate such damage through antioxidant defense systems, including the ascorbate–glutathione (AsA–GSH) cycle, in which ascorbate serves as an electron donor for detoxification of H_2_O_2_. Antioxidant enzymes such as APX, GPX, and GR play critical roles in this process. GR catalyzes the NADPH-dependent reduction in oxidized glutathione (GSSG) to its reduced form (GSH), thereby maintaining a high GSH/GSSG ratio. Our results indicate that the T_1_ transgenic lines exhibited significantly higher activities of antioxidant enzymes—including APX, GPX, and GR—under salt stress compared with control plants. These findings suggest that the overexpression of *PEPCK* is associated with modulating the antioxidant and defense machinery in rice under saline conditions, along with enhanced ROS-scavenging capacity.

Sugars also play an important role in plant defense against salinity by stabilizing cellular structures, interacting with phospholipid head groups, and contributing to ROS detoxification [42]. In this study, transgenic lines overexpressing *PEPCK* accumulated higher levels of fructose and glucose compared with control plants. The elevated accumulation of these sugars is suggestive of an enhanced capacity for osmotic adjustment and energy availability under saline conditions. Although the core metabolic mechanisms were not specifically investigated in the present investigation, the findings offer insightful information about their possible contribution. Similar accumulation of soluble sugars under salt stress has been reported in several plant species, including *Medicago sativa*, *Z. mays*, and *Vitis vinifera* [43,44,45]. Moreover, the transgenic rice plants showed significantly higher endogenous levels of plant hormones in both shoots and roots. These elevated hormone levels may have contributed to improved growth, cellular expansion, regulation of molecular and biochemical pathways, as well as stress adaptation in the transgenic plants under salt stress. However, further research is required in order to determine their specific regulatory mechanisms. These observations are consistent with previous reports highlighting the role of phytohormones in stress adaptation [46,47,48].

**Figure 6:**
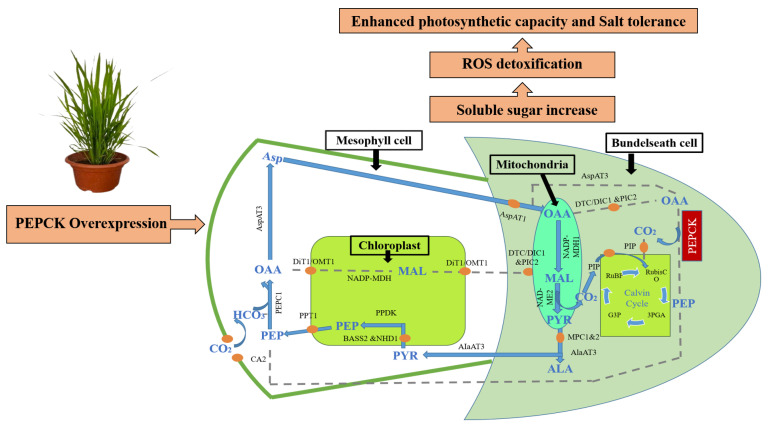
Schematic representation of biochemical and physiological responses of plants under salinity stress and how to counteract these effects. The plants activate upregulation of phosphoenolpyruvate carboxykinase (*PEPCK*), which promotes gluconeogenesis and modulates the tricarboxylic acid (TCA) cycle. This metabolic adjustment leads to increased soluble sugar accumulation and enhanced reactive oxygen species (ROS) detoxification, ultimately contributing to enhancing photosynthetic capabilities and improved stress tolerance. Abbreviation: CO_2_ carbon dioxide; HCO_3_^−^: bicarbonate; OAA: oxaloacetic; MAL: malate; PYR: pyruvate; Asp: aspartate and PEP: phosphoenolpyruvate. Names in black correspond to C4 genes; CA: carbonic anhydrase; PEPC: phosphoenolpyruvate carboxylase; AspAT: aspartate aminotransferase; DiT1: dicarboxylate transporter; OMT1: oxoglutarate/malate transporter; NADP-MDH: NADP-dependent malate dehydrogenase; DTC: dicarboxylate/tricarboxylate transporter; DIC1: dicarboxylate carrier; PIC: phosphate carrier; NAD-ME: NAD-malic enzyme; PEPCK: phosphoenolpyruvate carboxykinase; PIP: plasma membrane intrinsic protein; MPC1&2: mitochondrial pyruvate carrier; AlaAT: alanine aminotransferase; PPDK: pyruvate phosphate dikinase; BASS2: sodium-dependent pyruvate transporter; NHD1 sodium: proton antiporter and PPT: phosphate/phosphoenolpyruvate translocator.

Based on these observations, we propose a conceptual model describing the role of *PEPCK* in salinity tolerance. Under high salinity conditions, excessive accumulation of Na^+^ ions disrupts cellular homeostasis and reduces photosynthetic efficiency. Overexpression of *PEPCK* is linked to variations in the metabolism of carbon, leading to increased production of osmoprotective sugars. However, this research did not explicitly evaluate the direct role of gluconeogenesis and the tricarboxylic acid (TCA) cycle, indicating that it is still theoretical. These metabolic adjustments, specifically the conversion to PEP from OAA via PEPCK and the subsequent alteration in carbon flux, help to maintain carbon–nitrogen balance, stabilize cellular pH, facilitate detoxification of ROS, and ultimately improve salinity tolerance (Figure 6). Figure 6 also depicts that, as *PEPCK* is involved in the decarboxylation step of the C4 pathway, it may serve as a link between the carbon metabolism and the stress-responsive metabolic process.

In conclusion, the present study demonstrates that overexpression of *PEPCK* enhances salinity tolerance in transgenic rice while maintaining growth and yield-related traits. These findings highlight the potential of targeting metabolic pathway components such as *PEPCK* to improve crop resilience to salinity stress and contribute to sustainable agricultural productivity under changing climatic conditions. Although the results are promising, it is important to recognize some limitations of our study. Adding to these significant findings, future investigations may broaden the relevance of our study. As an example, assessing *PEPCK*-overexpressing lines in a wider range of field-specific saline environments would offer additional insight into how well they function in varying environmental situations. Furthermore, if a greater number of independent transgenic lines and a variety of genetic backgrounds are included, then the findings would have broader applicability. Although the current work indicates that *PEPCK* may be involved in controlling antioxidant defense, osmotic balance, and carbon metabolism at the mechanistic level, it remains to be further elucidated. Future research may provide insight into *PEPCK*’s role in pathways, including gluconeogenesis and the TCA cycle, using sophisticated techniques like metabolic flux measurement, isotope labeling, and thorough enzymatic investigations. Further comprehensive research might help create more effective methods for designing crops to withstand salt and expand our knowledge of *PEPCK*-mediated stress responses.

## Figures and Tables

**Table 1:** Agronomical parameters of rice (*O. sativa* L. cv. IR64) null-segregant and T_1_ generation of *PEPCK* overexpressing transgenic lines (line 2, line 7, and line 12) under 0 and 200 mM NaCl on 60 day old plant (salt treatment was given for 30 days).

Attributes	Control Plants		NaCl (mM)-Grown T_1_ *PEPCK* Transgenic Plants					
			L2		L7		L12	
	0	200	0	200	0	200	0	200
**Plant height (cm)**	72 ± 3.0 ^b^	31 ± 2.0 ^c^	76 ± 2.6 ^a^	68 ± 2.6 ^b^	71 ± 3.0 ^b^	66 ± 3.6 ^b^	78 ± 2.0 ^a^	71 ± 3.0 ^b^
**Root length (cm)**	26 ± 1.0 ^ab^	12.3 ± 1.1 ^c^	28.3 ± 0.5 ^a^	23.1 ± 0.7 ^b^	23.8 ± 0.7 ^b^	23 ± 0.5 ^b^	29.6 ± 0.7 ^a^	25.1 ± 1.0 ^ab^
**Root dry weight (g)**	2.5 ± 0.4 ^b^	1.3 ± 0.2 ^c^	2.8 ± 0.1 ^a^	2.0 ± 0.2 ^b^	3.1 ± 0.3 ^a^	2.5 ± 0.4 ^b^	2.6 ± 0.3 ^b^	2.3 ± 0.1 ^b^
**Leaf area (cm^2^/plant)**	93 ± 3.6 ^ab^	39 ± 3.0 ^c^	93 ± 2.5 ^ab^	84 ± 3.0 ^b^	97 ± 2.0 ^a^	92 ± 2.5 ^ab^	100 ± 2.6 ^a^	87 ± 2.0 ^b^
**Total protein (mg g^−1^ FW)**	1.9 ± 0.04 ^a^	0.7 ± 0.11 ^c^	2.0 ± 0.10 ^a^	1.5 ± 0.22 ^b^	2.0 ± 0.09 ^a^	1.9 ± 0.10 ^ab^	1.9 ± 0.05 ^a^	1.6 ± 0.26 ^b^
**Nitrogen (%)**	0.3 ± 0.006 ^b^	0.1 ± 0.004 ^c^	0.3 ± 0.012 ^a^	0.3 ± 0.014 ^b^	0.3 ± 0.015 ^a^	0.3 ± 0.009 ^ab^	0.3 ± 0.010 ^a^	0.3 ± 0.011 ^b^
**Phosphorus (%)**	0.3 ± 0.008 ^a^	0.1 ± 0.008 ^c^	0.3 ± 0.009 ^ab^	0.2 ± 0.009 ^b^	0.3 ± 0.009 ^a^	0.2 ± 0.008 ^b^	0.3 ± 0.006 ^ab^	0.2 ± 0.007 ^b^
**Potassium (%)**	0.2 ± 0.001 ^a^	0.1 ± 0.007 ^c^	0.2 ± 0.008 ^a^	0.1 ± 0.006 ^b^	0.2 ± 0.004 ^a^	0.1 ± 0.002 ^b^	0.1 ± 0.003 ^b^	0.1 ± 0.004 ^b^
**Sodium (%)**	0.04 ± 0.003 ^b^	0.07 ± 0.002 ^a^	0.04 ± 0.004 ^b^	0.05 ± 0.005 ^b^	0.04 ± 0.004 ^b^	0.04 ± 0.003 ^b^	0.04 ± 0.004 ^b^	0.07 ± 0.003 ^a^

Each value represents mean of three replicates ± SE. Means were compared using ANOVA. Different letters on the top of bars indicate significant differences at *p* ≤ 0.05 level, as determined by Duncan’s multiple range test (DMRT).

**Table 2:** Comparison of various yield parameters in rice (*O. sativa* L. cv. IR64) control and T_1_ generation of *PEPCK* overexpressing transgenic lines on 90 day old plant (line 2, line 7 and line 12) under 0 and 200 mM NaCl.

Parameters	Control Plants		NaCl (mM)-Grown T_1_ *PEPCK* Transgenic Plants					
			L2		L7		L12	
	0	200	0	200	0	200	0	200
**Time required for flowering (days)**	96 ± 3.6 ^a^	ND	95 ± 3.6 ^a^	96 ± 3.9 ^a^	97 ± 3.0 ^a^	97 ± 3.6 ^b^	92 ± 3.6 ^a^	95 ± 2.2 ^a^
**No. of tillers/plant**	21 ± 1.52 ^b^	ND	24 ± 0.17 ^a^	18 ± 0.57 ^c^	26 ± 0.57 ^a^	20 ± 0.17 ^b^	22 ± 1.15 ^ab^	14. ± 0.57 ^c^
**No. of** **panicle/plant**	25 ± 1 ^a^	ND	23 ± 0.6 ^ab^	14 ± 0.5 ^c^	26 ± 0.6 ^a^	17 ± 1.0 ^b^	20 ± 0.5 ^b^	11 ± 0.57 ^c^
**No. of filled grain/panicle**	86 ± 3.4 ^b^	ND	89 ± 3.0 ^ab^	80 ± 3.0 ^c^	94 ± 3.0 ^a^	83 ± 3.0 ^b^	84 ± 3.6 ^b^	72 ± 3.6 ^c^
**No. of chaffy grains/panicle**	10.3 ± 0.57 ^a^	ND	5.3 ± 0.57 ^c^	10.6 ± 0.57 ^a^	7.6 ± 0.57 ^b^	8.6 ± 0.57 ^b^	4.6 ± 0.57 ^c^	11 ± 1.0 ^a^
**Straw dry weight (g)**	55 ± 3.0 ^b^	ND	59 ± 2.6 ^ab^	49 ± 2.6 ^c^	63 ± 3.0 ^a^	54 ± 2.51 ^b^	59 ± 2.5 ^ab^	48 ± 1.15 ^c^
**100 grain weight**	2.9 ± 0.18 ^a^	ND	3.0 ± 0.30 ^a^	2.8 ± 0.13 ^c^	3.2 ± 0.15 ^a^	2.6 ± 0.06 ^b^	2.5 ± 0.13 ^b^	2.2 ± 0.06 ^c^

Control plants did not survive until harvesting under 200 mM NaCl. Each value represents mean of three replicates ± SE. Means were compared using ANOVA. Different letters on the top of bars indicate significant differences at *p* ≤ 0.05 level as determined by Duncan’s multiple range test (DMRT). ND: not determined as the control plants did not survive or had poor growth under salt stress conditions.

**Table 3:** Segregation ratio (Hyg^R^:Hyg^S^) of T_1_ *PEPCK* overexpressing transgenic rice plants (*O. sativa* L. cv. IR64).

	WT (Control)	Line 2	Line 7	Line 12
Segregation ratio (Hyg^R^:Hyg^S^) [n] ^a^	0	2.62:1 [178]	2.72:1 [156]	3.1:1 [184]

^a^ Recording made from seeds.

## Data Availability

The original contributions presented in this study are included in the article. Further inquiries can be directed to the corresponding authors.
